# Usefulness of Intravenous Gadolinium Inner Ear MR Imaging in Diagnosis of Ménière’s Disease

**DOI:** 10.1038/s41598-018-35709-5

**Published:** 2018-12-03

**Authors:** Young Sang Cho, Jung Min Ahn, Ji Eun Choi, Hyun Woo Park, Yi-Kyung Kim, Hyung-Jin Kim, Won-Ho Chung

**Affiliations:** 1 Department of Otorhinolaryngology-Head and Neck Surgery, Samsung Medical Center, Sungkyunkwan University School of Medicine, Seoul, Korea; 2 Department of Otorhinolaryngology-Head and Neck Surgery, Dankook University Hospital, Cheonan, Korea; 3 Department of Otorhinolaryngology-Head and Neck Surgery, Gyeongsang National University Hospital, Jinju, Korea; 4 Department of Radiology, Samsung Medical Center, Sungkyunkwan University School of Medicine, Seoul, Korea

## Abstract

This study aimed to investigate the usefulness of the intravenous gadolinium enhanced inner ear magnetic resonance imaging (IV-Gd inner ear MRI) in diagnosing Ménière’s disease(MD) and find a correlation between the degree of endolymphatic hydrops(EH) and the audiovestibular tests. Total 29 patients diagnosed with unilateral definite MD were enrolled. All patients underwent IV-Gd inner ear MRI and auditory and vestibular function tests such as pure tone audiometry (PTA), electrocochleography (ECoG), cervical vestibular evoked myogenic potential (cVEMP) and caloric test. The hydrops ratio in the cochlea and vestibule were significantly higher in the affected side than the unaffected side (*p* < 0.001). Average pure-tone thresholds for 0.5, 1 k, 2 k, and 4 k Hz correlated significantly with cochlear and vestibular hydrops (*p* < 0.01) in the affected side. When comparing the SP/AP ratio of ECoG with hydrops ratio in the vestibule, the affected and unaffected ears showed a significant difference (*p* < 0.05). Similarly, the results of the caloric test also showed a significant correlation (*p* < 0.05) with relative vestibular hydrops. However, the cVEMP response was not related to the hydrops ratio in the cochlea or vestibule. This study presents pertinent data with appropriate correlations with auditory vestibular functional testing which demonstrates the usefulness of IV-Gd inner ear MRI as a diagnostic method for visualizing the endolymphatic hydrops in MD.

## Introduction

Ménière’s disease (MD) is a multifactorial disorder in which typical symptoms include recurrent episodes of vertigo, fluctuating hearing loss, tinnitus, and sensation of ear fullness. Endolymphatic hydrops (EH) has been a histologic hallmark of MD. According to the American Academy of Otorhinolaryngology and Head and Neck Surgery (AAO-HNS)^1^, certain diagnosis of MD is only possible if patients are histologically confirmed of EH after death^2^. In other words, the diagnosis of certain MD, which requires histological confirmation, is impossible for living patients.

However, with the introduction of the MRI technique, it has become possible to visualize EH in living patients. In 2007, Nakashima *et al*.^3^ reported the visualization of EH in patients with definite MD using inner ear MRI with intratympanic (IT)-Gd administration. Although IT-Gd enhanced inner ear MRI has been able to identify hydrops from living patients directly, there are several disadvantages. It is an invasive technique and requires an additional procedure to visualize both sides. Additionally, the contrast Gadolinium is off-labeled for intratympanic use, and patients have to wait 24 hours after intratympanic injection^4^.

To overcome these shortcomings, intravenous (IV)-Gd enhanced inner ear MRI was developed by Naganawa *et al*.^5^ which visualized EH in patients with MD the same way as the IT technique while remedying the disadvantages of IT. The IV-Gd enhanced inner ear MRI is not invasive, have shorter waiting time (4 hours) and can visualize both inner ears simultaneously which enables identification of the asymptomatic EH in the opposite ear^6,7^.

After developments of inner ear MRI, either IT-Gd or IV-Gd enhanced, many efforts have been made to find a correlation between the endolymphatic hydrops and the test results of MD^8^. However, most of the studies used IT-Gd enhanced inner ear MRI, and the results also differed from study to study. In the case of IV-Gd enhanced inner ear MRI, a few studies^4,7,9,10^ have been conducted, and none of the studies compared widely with various audiovestibular tests. Therefore, comparing IV-Gd inner ear MRI with various objective audiovestibular tests and comparing results with IT-Gd inner ear MRI is a meaningful study.

The objective of this study was to find a correlation between the hydrops level in the patients with definitive MD using IV-Gd inner ear MRI and various audiovestibular tests (PTA, ECoG, VEMP, Caloric test). Moreover, these results were compared to similar published data performed with IT-Gd inner ear MRI. Ultimately, this study aimed to investigate the effectiveness of IV-Gd inner ear MRI in diagnosing MD.

## Methods

### Study Setting and Patients

Twenty-nine patients (19 men, 10 women, Mean age 48.9 yr) who were diagnosed with unilateral definite MD according to the revised diagnostic criteria by Classification Committee of the Bárány Society (2015)^11^ were recruited for inner ear MRI. The patients have experienced at least two episodes of vertigo attack lasting more than 20 minutes during the last three months. The patients who underwent surgical treatment or intratympanic gentamicin treatment for intractable vertigo were excluded from this study. Patients underwent PTA, ECoG, VEMP and caloric test. Written informed consent was obtained from all participants prior to conduct study. This study was approved by the institutional review board at the Samsung Medical Center following the declaration of Helsinki (IRB File No. 2014-08-101-007).

### Inner Ear MR Imaging

The inner ear MR imaging was performed on a 3.0-Tesla unit (MAGNETOM^®^ Skyra, Siemens medical solution, Erlangen, Germany) using a 32-channel array head coil. All patients waited 4 hours after a single dose (0.2 mL/kg or 0.1 mmol/kg body weight) IV administration of Gadobutrol (gadolinium-DO3A-butriol, Gadovist® 1.0; Schering, Berlin, Germany) before undergoing MR imaging. All patients underwent heavily T2-weighted (hT2W) MR cisternography (MRC) for anatomical reference of total endolymphatic fluid, hT2W– 3D-FLAIR with inversion time of 2250 ms (positive perilymph image, PPI), and hT2W–3D-IR with inversion time of 2050 ms (positive endolymph image, PEI) for evaluating endolymphatic hydrops.

Detailed parameters for MRC, PPI, and PEI were as follows. Parameters of MRC were: heavily T2-weighted MRC images using variable flip angles, 3D turbo spin echo technique (SPACE: sampling perfection with application-optimized contrasts by using different flip angle evolutions): repetition time (TR), 4400 ms; echo time (TE), 540 ms; initial refocusing flip angle (FA) of 180 degree rapidly decreased to constant FA of 120 degree for the turbo-spin-echo refocusing echo train, for the SPACE turbo spin- echo refocusing echo train; echo train length, 173 with restore magnetization pulse (fast recovery pulse); matrix size, 322 × 384; 104 axial slices of 1.0-mm thickness; field of view (FOV), 196 × 165 mm; generalized auto-calibrating partially parallel acquisition (GRAPPA) parallel imaging technique; acceleration factor, 2; number of excitations (NEX), 1.8; and scan time, 3 minutes 13 seconds.

Parameters of PPI were: SPACE sequence; TR, 9000 ms; TE, 540 ms; inversion time, 2250 ms; frequency-selective fat-suppression pre-pulse, initial refocusing FA of 180 degree rapidly decreased to constant FA of 120 degree for the turbo-spin-echo refocusing echo train; echo-train length, 173; matrix size, 322 × 384, and 104 axial 1.0-mm-thick slices covering the labyrinth with a 196 × 165 mm FOV; acquired using the GRAPPA parallel imaging technique with acceleration factor of 2; NEX, 4; and scan time, 14 minutes 51 seconds.

The parameters of PEI were the same as that of PPI except that the inversion time was 2050 ms. MR cisternography, PPI, and PEI employed identical FOV, matrix size, and slice thickness to facilitate comparison. We made HYDROPS (HYbriD of Reversed image Of Positive endolymph signal and native image of positive perilymph Signal) images on the scanner console by subtracting the PEI from the PPI. To increase the contrast-to-noise ratio of HYDROPS images, HYDROPS-Mi2 images were generated on a DICOM viewer (OsiriX MD image software, ver. 7.5.1 64 bit; Pixmeo Sarl, Bernex, Switzerland) by multiplication of HYDROPS and MRC images.

### Image Analysis

**Figure 1:**
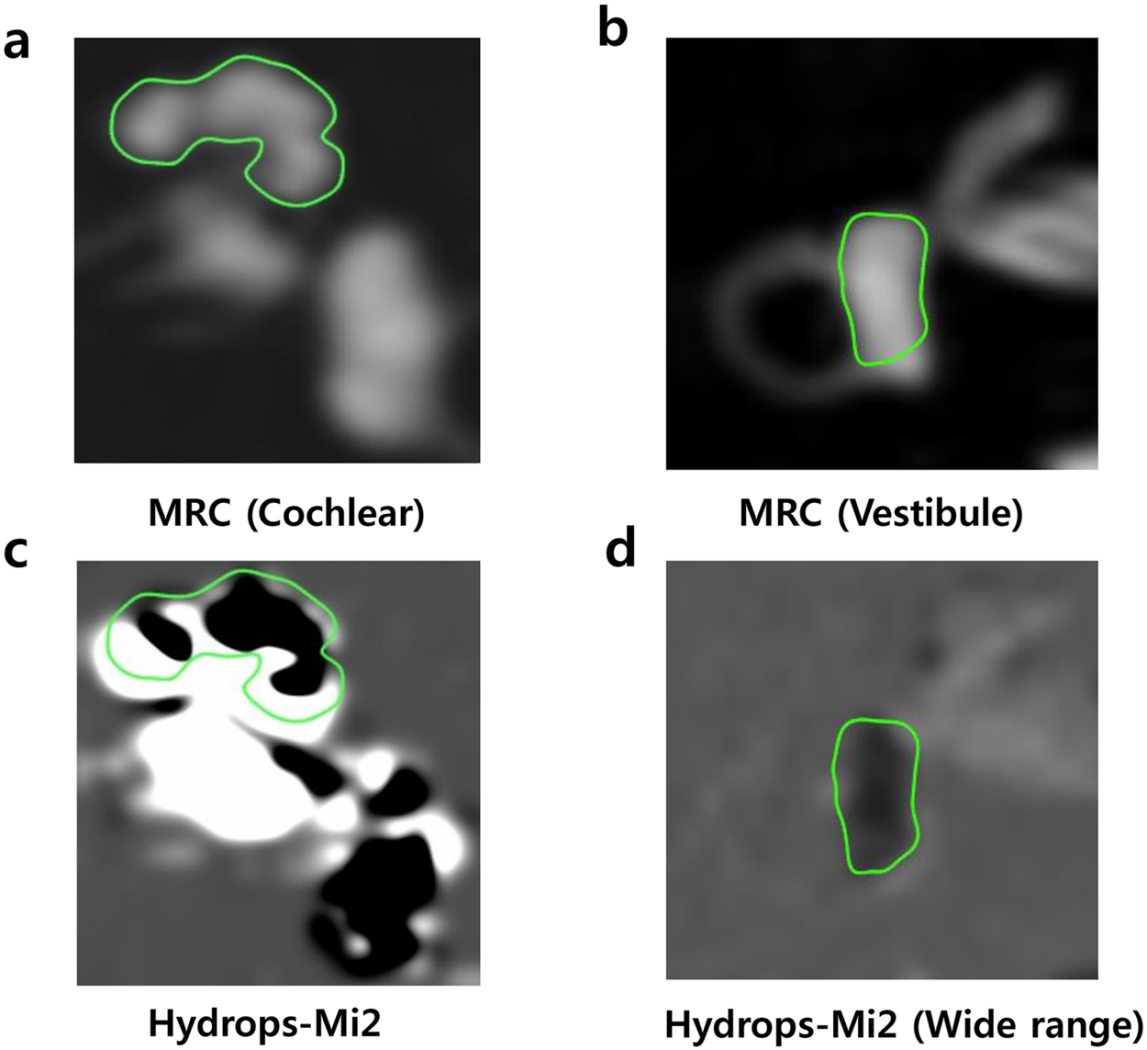
The process of setting the region of interest (ROI) to calculate the hydrops ratio. (**a**) In the MRC image, the ROI for the cochlear hydrops was drawn along the contour by selecting the slice that best showed the cochlear modiolus. (**b**) For the vestibular ROI, the lowest slice that the lateral semicircular canal was visualized more than 240° was selected, and the ampulla was excluded when drawing ROI on MRC. (**c**) A copy of the cochlear ROI from the MRC image was pasted to the HYDROPS-Mi2 image, and the hydrops ratio was calculated by dividing the number of pixels having negative pixels from the whole pixels. (**d**) A copy of the vestibular ROI from the MRC image was pasted in the HYDROPS-Mi2 (wide range) image. Vestibular hydrops with a negative signal inside is clearly observed.

One neuro-radiologist and two otologists independently evaluated the images. According to the methods proposed by Naganawa *et al*.^12^, each observer manually contoured the cochlea and vestibule separately to set up a region of interest (ROI) on the MRC after receiving the following instructions: (1) Before drawing the contour of the cochlea or vestibule margin on MRC, change the image window level and width to 400/1000 to get the best visual clarity. (2) For the cochlear ROI, select the slice where the cochlear turns (Basal, middle, apical) are visible. If every turn is visible on 2 or more slices, choose the slice with the largest height of the modiolus. (3) For the vestibular ROI, select the lowest slice the lateral semicircular canal ring is visualized more than 240° and exclude the ampulla when drawing ROI for the vestibule on MRC. These ROIs drawn on MRC were copied and pasted onto HYDROPS-Mi2 images (Fig. 1). Then used the histogram function of OsiriX to measure the numbers of pixels in the ROI and the numbers of pixels with negative signal intensity values (i.e., endolymph) in the ROI. The ratio of the area (z) of the endolymphatic space in the entire lymphatic space (zEL) was defined as zEL = (the number of negative pixels for the endolymph in the ROI divided by the total number of pixels in the ROI) × 100.

### Audio-Vestibular Tests: PTA, ECoG, VEMP, and Caloric Test

Every patient underwent PTA with 6 frequencies (0.25, 0.5, 1.0, 2.0, 4.0, 8.0 kHz) and the pure tone average was calculated from 4 frequencies (0.5, 1.0, 2.0, 4.0 kHz). PTA were divided into three subgroups as low (0.25 and 0.5 kHz), middle (1.0 and 2.0 kHz), and high (4.0 and 8.0 kHz) frequency.

Extra tympanic ECoG was performed bilaterally on each patient. ECoG was conducted using the Nicolet Viking IV (Nicolet, Madison, WI, USA) evoked potential averager. The summating potential (SP) was measured at the highest point of the shoulder of the ascending portion of the click-evoked response. The action potential (AP) amplitude was measured at the highest point of the response. A reproducible SP:AP ratio of greater than 34% was considered abnormal for the click stimulus^13^.

cVEMP was performed using Navigator Pro (Bio-logic Systems, Mundelein, IL, USA). Each subject was tested with cVEMP while holding their head away from the floor to contract the sternocleidomastoid muscle. Acoustic stimuli were presented through the inserted earphones. Acoustic stimuli of 500 Hz tone bursts was presented five times/second. To estimate the relative response in both ears, we used the interaural difference (IAD) ratio, calculated as (contralateral side ear amplitude-lesion side ear amplitude) ÷ (contralateral side ear amplitude + lesion side ear amplitude). Previously in our laboratory, we interpreted the VEMP results based on the absolute value of the IAD ratio from 33 normal subjects. The results revealed a mean ± SD IAD ratio of 12.72% ± 7.94%. A mean IAD ratio exceeding 30% indicated abnormal VEMP^14^.

The caloric test was performed using an infrared video-oculographic system (Micromedical Technologies, Chatham, Illinois, USA) and a Brookler–Grams closed-loop irrigation unit. Both ears were irrigated alternately with a constant flow of water at temperatures of 30 and 44 °C for 40 seconds. If the asymmetry between the responses for the affected and unaffected ear was >26%, the result was considered abnormal CP.

### Statistical Analysis

We used SPSS 18.0 software (SPSS Inc., Chicago, Illinois, USA) for all statistical analyses and adopted *P* value of <0.05 as the significance level for statistical testing.

Inter-observer reliability was calculated by using the intraclass correlation coefficient(Two way mixed, absolute agreement, ICC 3.3). To compare the cochlear hydrops (CH) and vestibular hydrops(VH) ratio between the affected and unaffected ear, *t*he t-test was used. The correlation between pure-tone thresholds and hydrops ratio was evaluated in Spearman correlation analysis. Also, we divided the patients into four stages based on pure-tone audiometry following the 1995 AAO-HNS guidelines to investigate the correlation between MD stage and hydrops ratio using ANOVA test.

We analyzed correlations between EH in the cochlea and vestibule, and ECoG, VEMP by using *t*-test. Correlation between caloric results and VH ratio was evaluated by comparing CP values to relative vestibular hydrops ratio (%RVH = [%VH affected ear − %VH unaffected ear]/[%VH affected ear + %VH unaffected ear]) using the Spearman correlation analysis. The Mann-Whitney test was used to compare CP values between the groups with and without lateral canal involvement. All tests were two-tailed, and *p*-values < 0.05 were considered significant.

## Results

Total 29 patients with definite MD underwent inner ear MR imaging with audiovestibular tests such as PTA, ECoG, c-VEMP and caloric test (Table 1).

**Table 1:** Clinical characteristics of the subjects enrolled in this study (N = 29).

No.	Sex	Age	MD side	Duration of disease(months)	PTA of the affected ear	Caloric test		ECoG of the affected ear	VEMP
						Weaker side	CP(%)	SP/AP ratio	IAD ratio
1	F	31	R	18	51.25	R	42	0.56	21*
2	M	63	R	22	66.25	R	24	NR	48
3	M	61	L	18	68.75	L	33	NR	11
4	M	21	R	7	22.5	L	14	0.27	9*
5	M	62	R	72	53.75	R	39	NR	10
6	M	53	R	18	72.5	R	58	NR	31
7	M	53	L	23	78.75	L	45	NR	ND
8	F	46	R	75	51.25	R	33	0.63	ND
9	M	46	L	7	30	L	13	0.31	15
10	F	34	L	80	35	L	50	0.23	24
11	M	27	R	27	41.25	L	55	0.41	33
12	M	65	R	53	55	R	0	0.45	8
13	M	52	L	132	72.5	L	55	NR	36
14	M	56	R	60	71.25	R	66	NR	ND
15	F	62	L	17	32.5	R	2	0.32	19
16	F	43	L	19	10	L	37	0.18	38
17	F	44	R	24	38.75	R	6	0.48	ND
18	M	20	R	44	45	R	8	0.67	33
19	M	42	L	18	72.5	L	100	NR	16
20	M	47	R	64	43.75	R	46	0.38	ND
21	M	59	R	31	46.25	L	7	0.28	31
22	M	55	R	50	58.75	R	96.3	NR	51
23	M	48	L	11	55	L	46	0.33	62
24	F	55	L	6	73.75	L	24	NR	10*
25	F	72	R	17	55	R	47	0.4	13
26	M	58	R	2	18.75	ND	ND	0.35	ND
27	M	52	L	49	56.25	ND	ND	0.52	ND
28	F	47	L	29	66.25	L	74	0.5	ND
29	F	46	R	65	35	R	100	0.31	58

ND, not done; NR, no response; *, unaffected ear.

### Interobserver Reliability

One neuro-radiologist and two otologists independently evaluated the images. Three raters analyzed the hydrops ratio of cochlear and vestibule in both ears without any clinical information (no knowledge of lesion side). Reliability analysis was performed between raters; the lowest coefficient exceeded 0.935, indicating excellent agreement between the raters.

### Visualization of Endolymphatic Hydrops

**Figure 2:**
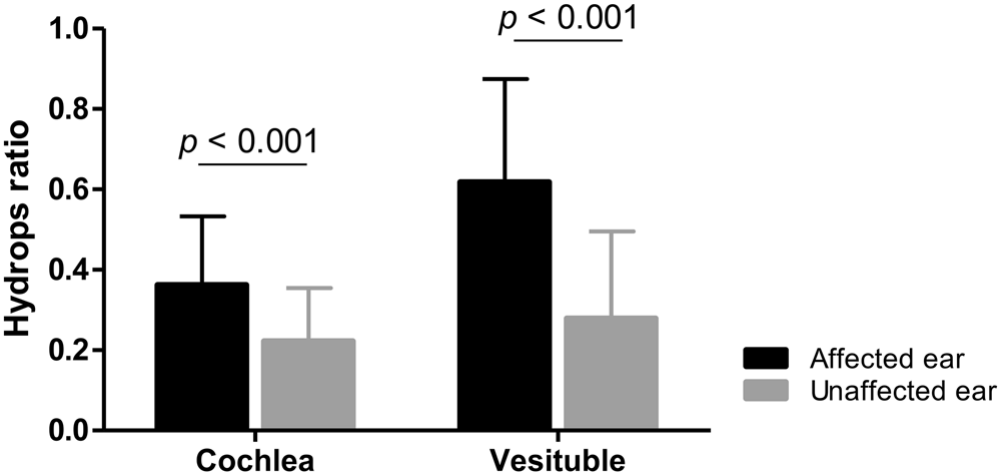
The ratios of cochlear and vestibular hydrops in MD patients. The ratios of cochlear and vestibular hydrops were significantly higher in the affected than the unaffected side. The mean ratio of endolymph in cochlea was 0.372 for the affected and 0.214 for the unaffected. Similarly, the mean ratio of endolymph in the vestibule was 0.667 for the affected and 0.266 for the unaffected.

We were able to visualize the EH clearly through IV-Gd inner ear MRI in 58 ears of 29 patients. The hydrops ratio between the affected and the unaffected side was compared. The hydrops ratios in the cochlea and vestibule (CH, VH) were significantly higher in the affected side than the unaffected side (*p* < 0.001). The mean hydrops ratio in the cochlea and vestibule was 0.372 and 0.667 for the affected side, and 0.214 and 0.266 for the unaffected side, respectively (Fig. 2).

### Pure Tone Audiometry (PTA) and the Hydrops Ratio of Cochlea and Vestibule

**Figure 3:**
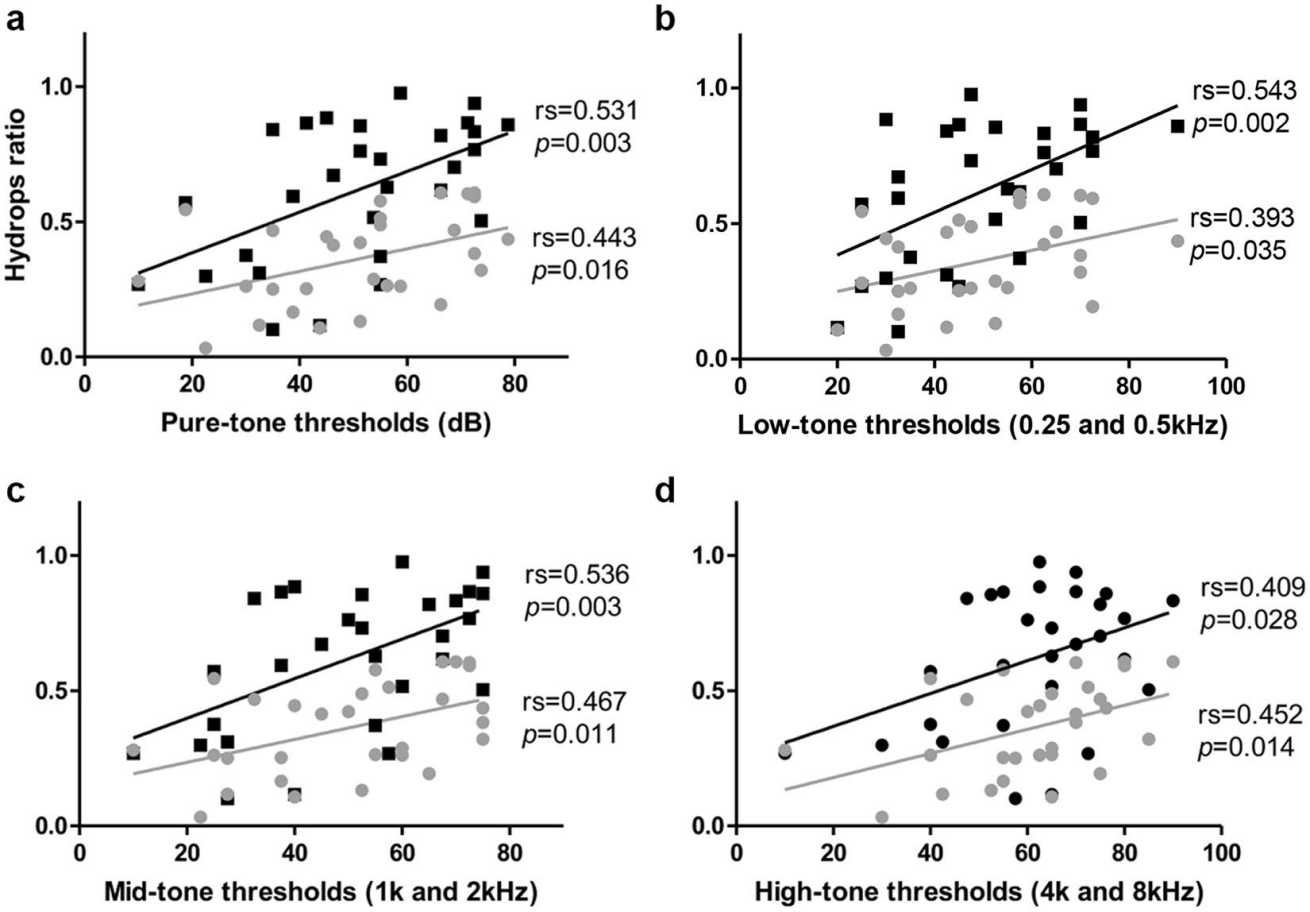
The correlation between the hydrops ratio and PTA. Air conduction 4-frequency pure tone average (500, 1000, 2000, and 4000 Hz PTA) was significantly correlated with EH ratio in cochlea (rs = 0.443, *p* = 0.016) and vestibule (rs = 0.531, *p* = 0.003) in affected ear. Additionally, hearing thresholds at low (250 and 500 Hz), mid (1000 and 2000 Hz), and high (4000 and 8000 Hz) frequency were significantly correlated with EH ratio.

**Figure 4:**
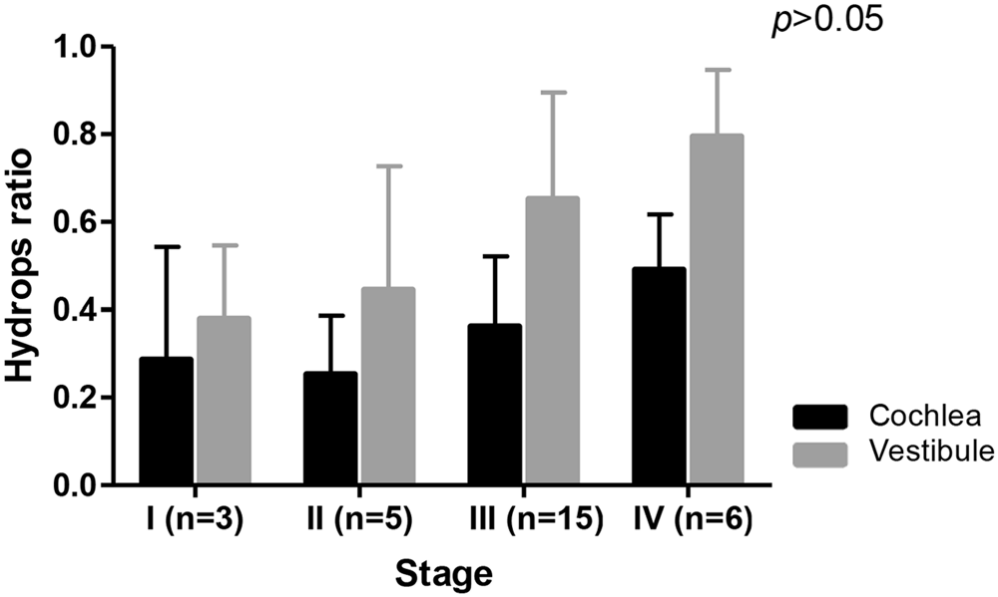
The correlation between the hydrops ratio and stages. Patients were divided into four stages according to the 1995 AAO-HNS guidelines. Overall, as the stage increased, the EH ratio tended to increase, but there was no significant difference between EH ratio and stages of MD.

The PTA of the 29 patients measured an average air conduction hearing threshold of 50.9 dB (±18.1) in the affected ears and 18.4 dB (±7.6) in the unaffected ears. Pure tone average for 0.5, 1 k, 2 k, and 4 k Hz correlated significantly with cochlear and vestibular EH ratio in the affected ear (*p* < 0.05). Additionally, hearing thresholds at low (250 and 500 Hz), mid (1000 and 2000 Hz), and high (4000 and 8000 Hz) frequency also had a significant correlation with EH ratio (Fig. 3). Patients were divided into four stages according to the 1995 AAO-HNS guidelines, as the stage increased, the CH and VH ratios tended to increase as well, but there was no statistical significance (Fig. 4).

### ECoG and the Hydrops Ratio of the Cochlea and Vestibule

**Figure 5:**
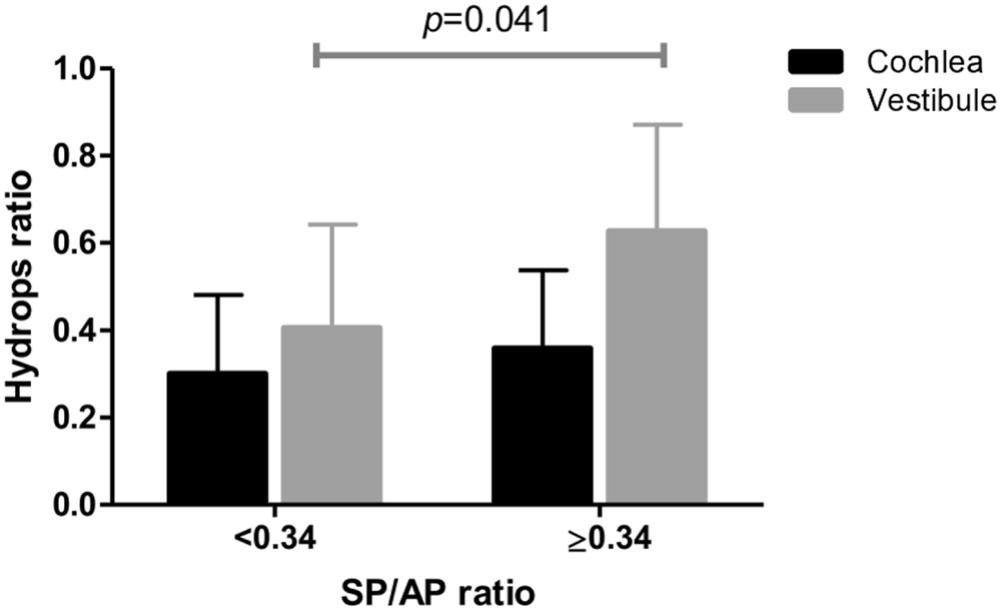
The correlation between the hydrops ratio and ECoG. Patients were divided into two groups according to the SP/AP ratio. Subjects with SP/AP ratio greater than 0.34 had significantly higher EH ratio in vestibule than those with SP/AP ratio less than 0.34 (*p* = 0.041).

The results of ECoG were divided into normal (SP:AP ratio < 0.34) and abnormal group (SP:AP ratio ≥ 0.34) accordingly. The hydrops ratio of the two groups was compared. Of the 29 patients, 10 had no response in the ECoG, and 11 of the remaining 19 patients showed an abnormal response in the ECoG. T testing between ECoG and IV-Gd inner ear MRI results showed significant (*p* < 0.05) difference in VH ratio between normal and abnormal ECoG in the affected ear (Fig. 5). However, there was no significant correlation between CH ratio and ECoG results in the affected ears.

### VEMP and the Hydrops Ratio of the Cochlea and Vestibule

**Figure 6:**
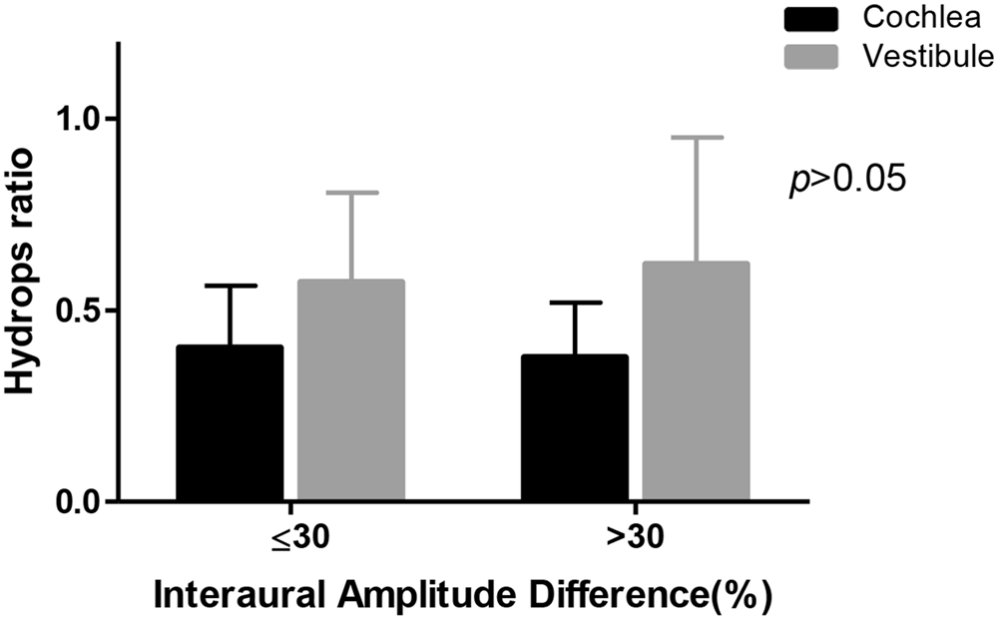
The correlation between the hydrops ratio and cVEMP. There was no significant difference of EH ratio in cochlea between two groups. In results of VEMP, the subjects with IAD greater than 30% tended to have higher EH in cochlea and vestibule. However, it was not statistically significant.

Twenty one out of the 29 patients who underwent cVEMP were divided into normal (n = 11, IAD ≤ 30%) and abnormal (n = 10, IAD > 30%) groups. The EH ratio of the two groups was compared, and there was no statistical significance between the two groups (Fig. 6).

### Caloric Test and the Hydrops Ratio of the Vestibule

**Figure 7:**
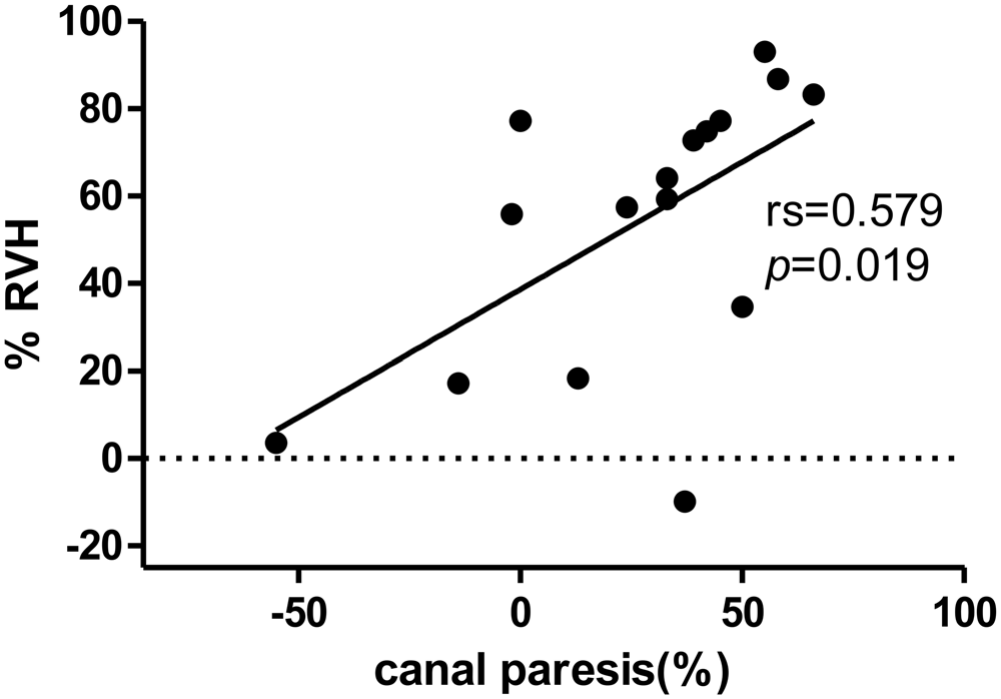
The correlation between the relative hydrops ratio and canal paresis. The value of canal paresis (CP) was significantly correlated with relative vestibular hydrops ratio (rs = 0.579, *p* = 0.019). %RVH = [%VH_affected ear_ − %VH_unaffected ear_]/[%VH_affected ear_ + %VH_unaffected ear_].

**Figure 8:**
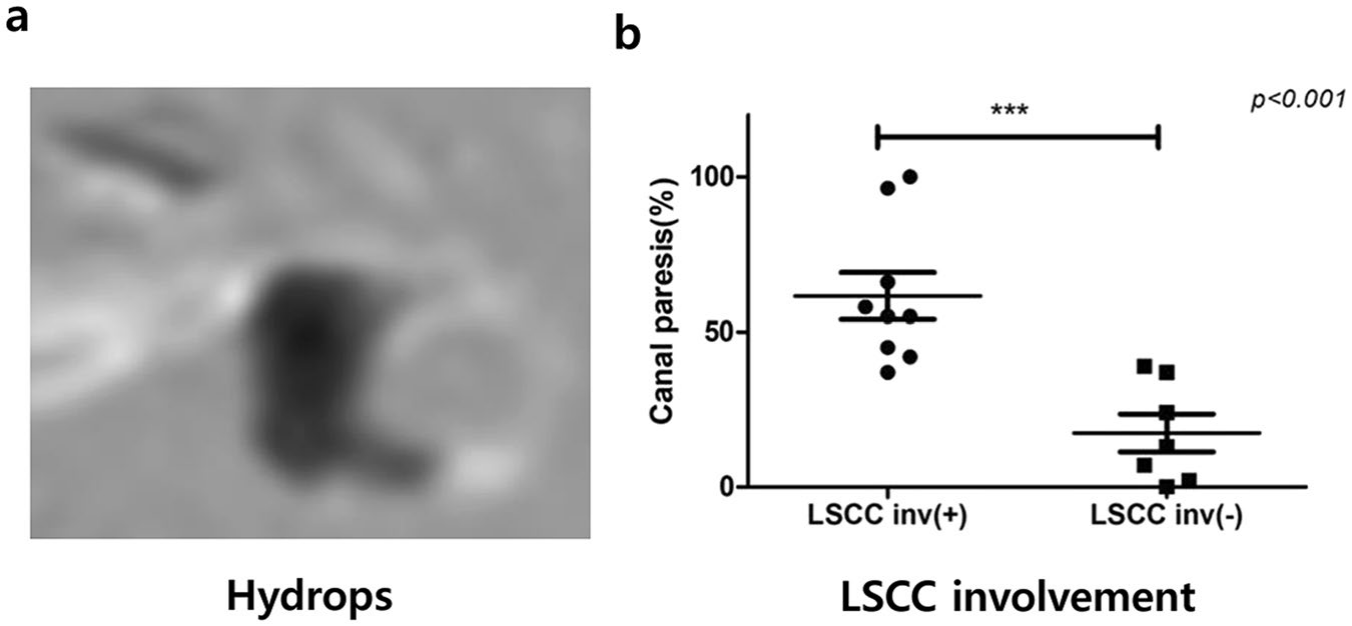
MR images of the vestibular hydrops involving the lateral semicircular canal. (**a**) This Hydrops image showed an extension of vestibular hydrops into the lateral semicircular canal. (**b**) The canal paresis in the patients with LSCC involvement was significantly higher than the patients without LSCC involvement (*p* < 0.001).

The caloric test was performed on 27 out of 29 patients. Of these, 11 patients were excluded from the analysis as the date of the MRI and the caloric test was more than six months apart. The cochlear and vestibular hydrops ratio were compared with the value of the canal paresis (CP). If the asymmetry between the responses for the affected and unaffected ear was >26%, the result was considered to be of abnormal CP. Eleven out of 16 patients (61%) showed abnormal CP. The mean value of CP in the abnormal group was 45.8 ± 11.2, and the mean value in the normal group was 12.3 ± 12.0. The relationship between relative VH ratio (%RVH) and caloric response for the affected ear is shown in Fig. 7. CP value correlated significantly with %RVH (*p* < 0.05). All the patients had normal vHIT. Vestibular hydrops was visualized extended into the lateral semicircular canal (LSCC) in 9 out of 16 (56.3%) patients (Fig. 8a). In particular, the CP value of the caloric test was significantly increased when the hydrops involved LSCC (Fig. 8b).

## Discussion

In this study, we visualized the endolymphatic hydrops (EH) in unilateral definitive MD patients using IV-Gd inner ear MRI. The hydrops ratios in the cochlea and vestibule were significantly higher in the affected side than the unaffected side. Besides, comparing the hydrops ratio with audiovestibular testing, the EH ratio had a significant correlation with audiovestibular tests such as PTA, ECoG and caloric test.

Ever since EH has been identified as a histopathological marker for patients with MD^15^, various efforts have been made to directly visualize EH in living patients to diagnose and evaluate MD. IT-GD inner ear MRI was first developed to visualize the cochleovestibular endolymphatic hydrops in living subjects^3^ and then the IV-Gd inner ear MRI, which improved upon the IT-Gd inner ear MRI by remedying the disadvantages, was introduced^16^.

Many reports have been published regarding the correlation between the various objective measures and inner ear MRI. It was summarized in Table 2. The hydrops level was mostly correlated with PTA and ECoG. However, correlation with VEMP and caloric test was variable^17^.

**Table 2:** Previous Studies Using IT or IV-Gd Enhanced Inner Ear MRI in MD.

Author	No. of participants	Type of disease	Injection route	Compared tests	Positive correlation findings with MRI
Gürkov *et al*.^25^	34	MD	IT	PTA, VEMP, Caloric test, ECoG	PTA, VEMP
Hornibrook *et al*.^33^	30	MD	IT	ECoG	ECoG
Sun *et al*.^34^	30	VM, MD	IT	PTA, VEMP	PTA, VEMP (partial)
Seo *et al*.^35^	26	MD	IT	PTA, ECoG, VEMP	PTA, ECoG:
Yamamoto *et al*.^36^	17	MD	IT	ECoG	ECoG
Fukuoka *et al*.^37^	20	MD	IT	ECoG	ECoG
Kato *et al*.^17^	24	MD	IT	Caloric test	
Fiorino *et al*.^26^	18	MD	IT	ECoG, VEMP, Caloric test	VEMP
Jerin *et al*.^38^	39	MD	IT	VEMP	
Sephadari *et al*.^9^	21	MD, delayed EH, SSNHL	IV	PTA	PTA
Quatre *et al*.^10^	41	MD	IV	PTA, ECoG, DPOAE, VEMP	PTA

Abbreviation: MD, Ménière’s disease; VM, Vestibular migraine; EH, Endolymphatic hydrops; SSNHL, Sudden sensorineural hearing loss; IT, Intratympanic; IV, Intravenous.

In our study, we collectively searched correlation of the hydrops level and the audiovestibular test results using IV-Gd inner ear MRI. This is the first study ever to comprehensively compare IV-Gd inner ear MR imaging with various audiovestibular tests. During 4 hours waiting time, other tests such as PTA, ECoG, caloric test, vHIT and VEMP were tested. It is a time-saving protocol for the patients. Besides, if the patients needed to exclude intracranial or cerebellopontine angle pathology, conventional contrast image was added immediately after contrast injection. It is noninvasive (not necessary to puncture the tympanic membrane), and enables to visualize both ears simultaneously and is not interfered by middle ear status to possibly compromise the round window or oval window permeability.

Using the methods that Naganawa *et al*. proposed^12^, we measured quantitatively the hydrops ratio in both cochlea and vestibule. There was a significant difference between the affected side and the unaffected side. The mean hydrops ratio in the cochlea and vestibule was 0.372 and 0.667 for the affected side, and 0.214 and 0.266 for the unaffected side. In the affected ear, the hydrops ratio in the vestibule was strongly correlated with the cochlear hydrops ratio.

When we analyzed the relationship between PTA and the hydrops ratio, there was a strong correlation between the hydrops ratio and PTA of whole frequencies. If we grouped into high, middle and low frequencies, there was also a strong correlation in each group. Although there was no statistical significance, as the MD stage increased, the ratio of CH and VH tended to increase generally. This supports that hearing loss got worse as the endolymphatic hydrops has progressed. These findings were similar to other reports using IT-Gd inner ear MRI (Table 2).

ECoG has been used for an adjunctive method to diagnose MD^18,19^. As SP is a DC potential, SP/AP ratio is elevated in endolymphatic hydrops. Because of the high sensitivity of SP/AP in MD, it has been used for years for the diagnosis of MD^20^. In our study, we found the VH was significantly larger in abnormal SP/AP (>0.34) rather than normal SP/AP. This meant that the severity of hydrops level is related with SP/AP. However, the hydrops level in the cochlea was not significantly different between the two groups, even though there was a tendency. These might be because of the limited number of patients. In the affected ear, mean cochlear hydrops ratio was smaller than the mean vestibular hydrops ratio (0.372 vs. 0.667). Since the mean value is low and the variance is small, it is estimated that the number of patients should be large to obtain a meaningful value.

Regarding cVEMP indicating the saccular function, there was no significant correlation between the hydrops level and interaural difference (IAD) ratio. Several studies have shown that cVEMP response was reduced or enhanced in MD depending on the duration and stage of the disease^21–24^. They suggested that enhanced cVEMP response in the affected ear was often associated with the early stage of MD, and as MD evolves, a decrease in amplitude was observed. In this study, the duration of the disease in patients with MD varied from 2 to 132 months, and we did not compare cVEMP according to the stage. Other reports were also variable in the relationship between the VEMP response and the EH ratio (Table 2). More investigation is needed to clarify the relationship between cVEMP and the hydrops ratio.

The value of CP in the caloric test was significantly correlated with relative VH ratio in the affected side. Several papers reported that there was no correlation between caloric response and hydrops level^17,25,26^. They included every MD patients regardless of vHIT results and did not consider the asymptomatic hydrops level in the opposite side^27,28^. Recently, a new mechanism for explaining the dissociation of caloric response and vHIT was proposed^29^. If the hydropic expansion of the endolymphatic duct in MD patients allows local flow within the duct, this could dissipate the hydrostatic pressure caused by the thermally-induced density difference and diminish or eliminate the deflection of the cupula. Thus, hydrops of endolymphatic duct could be the cause of caloric deficit without compromising the vestibulo-ocular reflex (VOR) response in vHIT. In the author’s other paper, we found out the morphological correlation between caloric tests and vestibular hydrops in MD patients with normal vHIT^30^. The patients with abnormal vHIT were excluded. And the hydrops level in the opposite side was considered by comparing the relative hydrops level. Therefore, the relative vestibular hydrops ratio showed the correlation with canal paresis in caloric tests.

For quantification of hydrops ratio, the HYDROPS-Mi2 image was used. ROI in the cochlea and vestibule was drawn manually on the MRC image as described in the methods. Selected ROIs on the MRC were copied and pasted onto HYDROPS-Mi2 image. Hydrops ratio in the cochlea and vestibule was automatically measured by calculating the ratio of the area of the endolymphatic space out of the entire lymphatic space using the histogram function of OsiriX. Therefore, the selection of ROI was the critical step to reduce the inter-observer variance.

In the clinical setting, the visual grading system proposed by Nakashima has been more commonly used^31^. It is easily interpreted by the radiologists. Also, it is faster without further processing. We also found out a significant correlation between the visual grading system and our quantitative method (data not shown). However, this visual grading system is so roughly divided by 3 (none, mild, significant) that it is not enough to estimate the hydrops ratio quantitatively. In addition, the quantitative measure of hydrops would be more valuable for the future research on MD.

The saccule to utricle ratio inversion (SURI) approach was introduced to grade the hydrops level in the 3D FLARE image^10,32^. But, even though the specificity was high, the saccular detection was difficult (especially in severe vestibular hydrops), and sensitivity was not high.

This study had some limitations. First, our study did not have a control group. As we made a comparison between the affected and unaffected ear, the hydrops ratio in the unaffected side did not mean the value of completely normal inner ear. As MD could be affected bilaterally, the normal value of hydrops ratio should be searched in the normal control.

Second, the duration of the disease was not considered in this study. MD is a long- standing disease. The hydrops level would be changed according to the disease duration. If the hydrops level in MRI is to be utilized for the diagnosis of MD, we should get the data of hydrops level according to the duration of disease.

Third, the number of enrolled patients was limited. With our quantification method, we manually drew the contour of the cochlea and vestibule on the MRC image. The region of interests (ROI) in the vestibule was easily drawn on MRC, and the vestibule in all parts could be occupied by the hydrops to varying degrees. However, the ROI in the cochlea included the areas where the hydrops could not involve such as modiolus and scala tympani. Therefore, the hydrops ratio in the vestibule was higher and more reliable than in the cochlea (0.667 vs. 0.372), even though these cochlear and vestibular hydrops ratios had a strong correlation. Additionally, we need to increase the number of patients and normal control to have a cutoff value of hydrops ratio in the vestibule and cochlea, especially in the cochlea.

In our study, IV-Gd MRI was able to measure the hydrops ratio of the cochlea and vestibule without any significant difference between examiners. And we confirmed that among the audiovestibular tests, PTA, ECoG and caloric tests were significantly correlated with the degree of vestibular hydrops. These tests might indicate the severity of hydrops in MD patients. Besides, IV-Gd MRI gives more benefit to the patients than IT-Gd MRI. It is a non-invasive technique with shorter waiting time, not interfered with by middle ear status and able to scan both ears and intracranial pathology simultaneously.

In conclusion, endolymphatic hydrops in the cochlea and vestibule are readily visualized using IV-Gd MRI. Hydrops image by IV-Gd MRI may be a reliable means to diagnose MD. Several audiovestibular tests (PTA, ECoG, caloric test) was correlated with severity of hydrops. We are confident that this method will reveal the pathophysiology of MD as well as help to better diagnose MD.
